# Rapid diagnosis of fatal *Nocardia kroppenstedtii* bacteremic pneumonia and empyema thoracis by next-generation sequencing: a case report

**DOI:** 10.3389/fmed.2023.1226126

**Published:** 2023-07-18

**Authors:** Fanfan Xing, Yao Xia, Qianyun Lu, Simon K. F. Lo, Susanna K. P. Lau, Patrick C. Y. Woo

**Affiliations:** ^1^Department of Clinical Microbiology and Infection Control, The University of Hong Kong—Shenzhen Hospital, Shenzhen, China; ^2^Department of Microbiology, Li Ka Shing Faculty of Medicine, The University of Hong Kong, Pokfulam, Hong Kong SAR, China; ^3^Doctoral Program in Translational Medicine and Department of Life Sciences, National Chung Hsing University, Taichung, Taiwan; ^4^The iEGG and Animal Biotechnology Research Center, National Chung Hsing University, Taichung, Taiwan

**Keywords:** *Nocardia kroppenstedtii*, immunocompromised patients, next-generation sequencing, Oxford Nanopore, MinION

## Abstract

*Nocardia* species do not replicate as rapidly as other pyogenic bacteria and nocardial infections can be highly fatal, particularly in immunocompromised patients. Here, we present the first report of fatal *Nocardia kroppenstedtii* bacteremic pneumonia and empyema thoracis diagnosed by next-generation sequencing (NGS) using the Oxford Nanopore Technologies' MinION device. The bacterium was not identified by matrix-assisted laser desorption ionization-time of flight mass spectrometry. Due to its low equipment cost, short turn-around-time, and portable size, the Oxford Nanopore Technologies' MinION device is a useful platform for NGS in routine clinical microbiology laboratories.

## Introduction

*Nocardia* species are ubiquitous environmental saprophytes. Human infections are often acquired through inhalation of the bacterium or direct inoculation of the skin. Respiratory and disseminated infections occur mostly in immunocompromised hosts, such as patients on high-dose corticosteroids, organ transplant recipients, and HIV-positive patients. A surge in cases of *Nocardia* infection has been witnessed in the last 20 years as a result of an increased number of immunocompromised patients. Moreover, there has also been a marked increase in the number of both known and novel *Nocardia* species associated with human infections because of the use of 16S rDNA gene sequencing and whole genome sequencing (1). Examples of novel *Nocardia* species discovered in recent years associated infections include *Nocardia guangzhouensis, Nocardia barduliensis, Nocardia gipuzkoensis*, and *Nocardia kroppenstedtii* (2–4).

Laboratory diagnosis of *Nocardia* infection relies largely on the isolation of the bacterium in clinical samples and accurate identification. Species identification of *Nocardia* was traditionally performed by biochemical tests but was time-consuming and unreliable. 16S rDNA gene sequencing is currently the working standard for the identification of *Nocardia* species. However, performing 16S rDNA gene sequencing in-house would require an expensive Sanger DNA sequencer, which is not readily available in most clinical microbiology laboratories. Matrix-assisted laser desorption ionization-time of flight mass spectrometry (MALDI-TOF MS) has been reported to be an alternative and reliable method for the identification of *Nocardia* species in some reports (5). However, accurate identification by MALDI-TOF MS depends on a good database, which is often not perfect, particularly for uncommon bacterial species (6, 7). With the more widespread use of next-generation sequencing (NGS), especially with the recent invention of the Oxford Nanopore Technologies' MinION device, 16S rDNA gene amplification coupled with NGS using the Oxford Nanopore Technologies' MinION device can be performed in-house without the purchase of the expensive Sanger DNA sequencing equipment. In this study, we describe a patient with *N. kroppenstedtii* bacteremia, pneumonia, and empyema thoracis diagnosed using the Oxford Nanopore Technologies' MinION device.

## Case description

**Figure 1:**
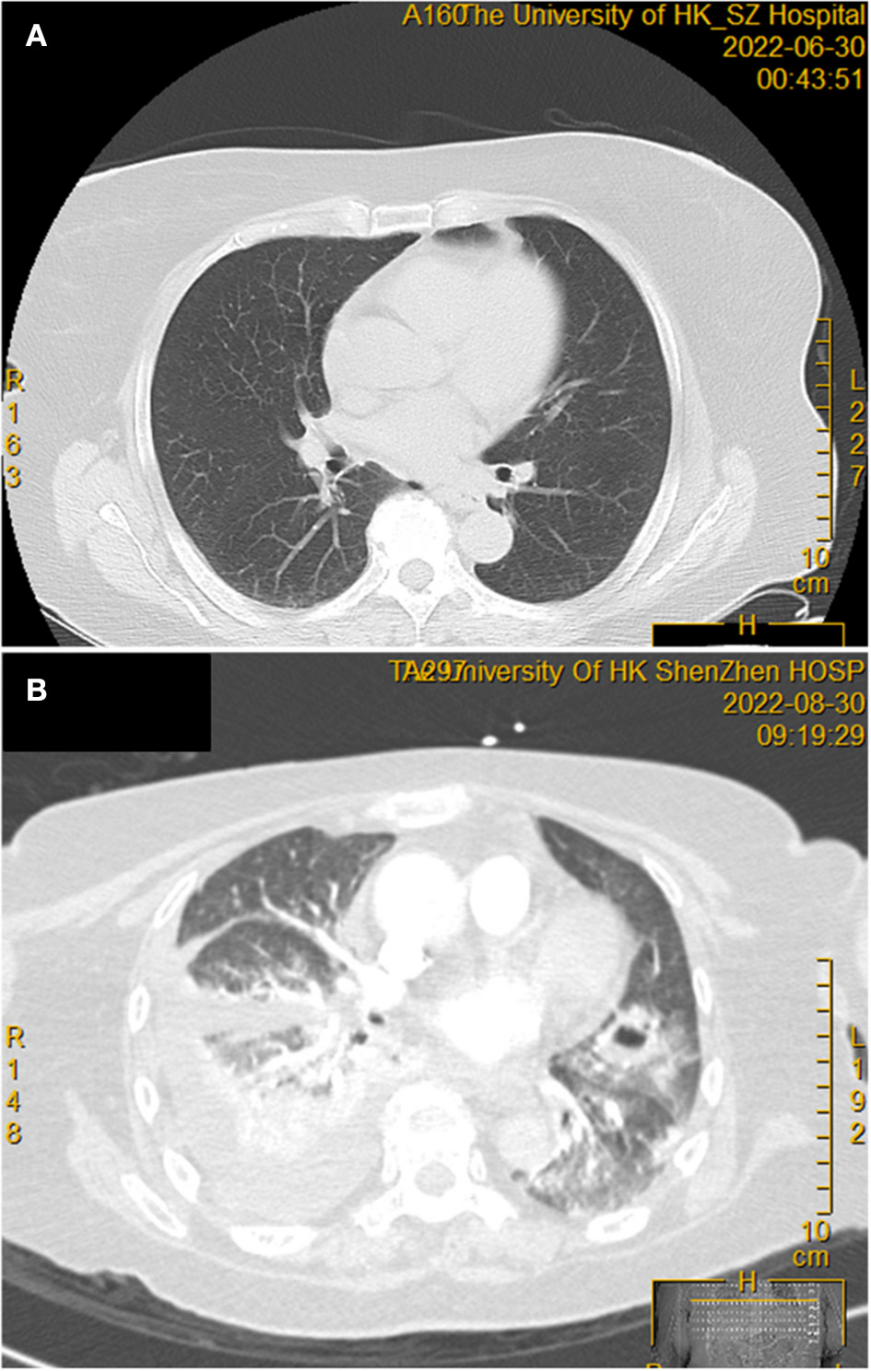
Thoracic computerized tomography scan before and after corticosteroid treatment. **(A)** No obvious abnormal tissue density shadow was observed before the commencement of the corticosteroid. **(B)** Two months after the commencement of a high dose corticosteroid, showing the new onset of right pleural effusion with increased density of adjacent lung parenchyma, as well as patchy infiltrates and cavitation in both lungs.

**Figure 2:**
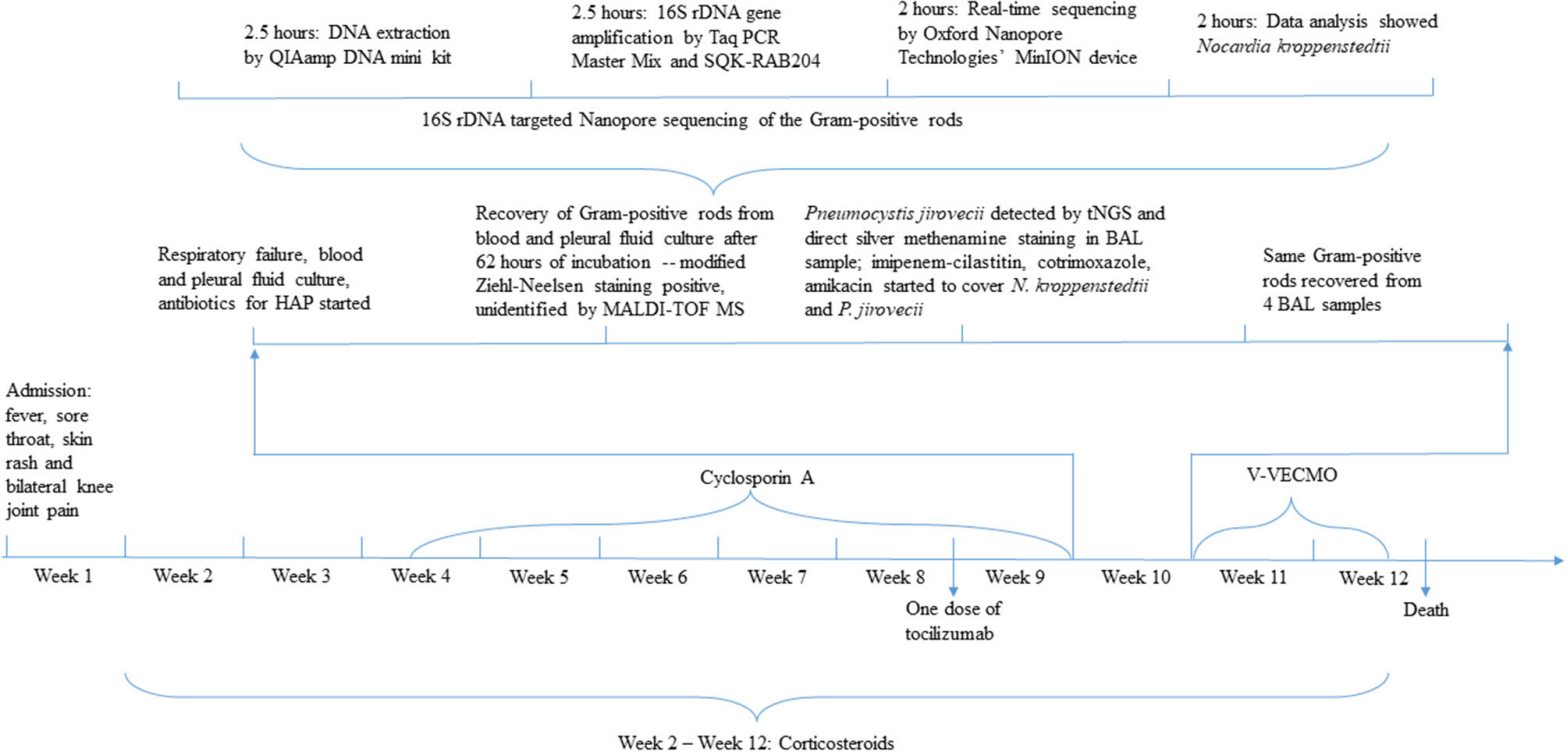
Timeline depicting rapid diagnosis of *Nocardia kroppenstedtii* infection by tNGS. HAP, hospital-acquired pneumonia; MALDI-TOF MS, matrix-assisted laser desorption ionization-time of flight mass spectrometry; tNGS, target next-generation sequencing; BAL, bronchoalveolar lavage; V-VECMO, Vein-vein extracorporeal membrane oxygenation.

A 71-year-old Chinese woman presented to our hospital because of progressive onset of fever, sore throat, maculopapular skin rash, and bilateral knee joint pain for 3 days. Blood results are shown in Table 1. Empirical intravenous ceftriaxone was prescribed but there was no improvement. Three sets of blood cultures yielded negative results. A computed tomography (CT) scan of the thorax and abdomen did not reveal any significant changes at that time (Figure 1A). Adult Still's disease was suspected and a high dose corticosteroid was commenced (Figure 2). Initially, the patient responded to the corticosteroid and so did the C-reactive protein and serum ferritin levels. However, the fever and other symptoms relapsed when the dose of corticosteroid was reduced and therefore the dose was increased again. Three weeks after disease onset, cyclosporine A was given for a total of 38 days. Eight weeks after disease onset, one dose of tocilizumab was prescribed.

**Table 1:** Blood results of the patient.

**Laboratory test**		**Results**		**Normal range**
		**On admission**	**Week 9**	
Complete blood counts	WBC (/L)	25.3 × 10^9^	10.02 × 10^9^	3.89–9.93 × 10^9^
	Neutrophil (/L)	22.37 × 10^9^	9.04 × 10^9^	2.01–7.42 × 10^9^
	Lymphocyte (/L)	1.54 × 10^9^	0.71 × 10^9^	1.06–3.61 × 10^9^
	Monocyte (/L)	1.37 × 10^9^	0.25 × 10^9^	0.18–0.65 × 10^9^
	Platelet (/L)	149 × 10^9^	50 × 10^9^	162–341 × 10^9^
	Hemoglobulin (g/L)	142	171	133–171
Alanine aminotransferase (U/L)		21.9	143.1	0–41
Aspartate aminotransferase (U/L)		47.1	44.7	0–40
Total bilirubin (μmol/L)		42.3	30.2	0–21
Albumin (g/L)		41.5	31.9	35–52
C-reactive protein (mg/L)		184.43	14.1	< 10
Procalcitonin (ng/mL)		0.6	1.53	< 0.25
Ferritin (ng/mL)		9,636	1,140.2	11–306.8
Arterial blood gas analysis	pH	Not tested	7.423	7.35–7.45
	Partial pressure of arterial carbon dioxide (kPa)	Not tested	4.02	4.67–6.40
	Partial pressure of arterial oxygen (kPa)	Not tested	8.6	11.1–14.4
	Oxyhemoglobin saturation	Not tested	92.70%	95–99%
	Lactic acid (mmol/L)	Not tested	6	0.5–1.6
	Actual bicarbonate concentration (mmol/L)	Not tested	19.3	21–28
	Standard bicarbonate concentration (mmol/L)	Not tested	21.4	22.5–26.9
	Standard base excess (mmol/L)	Not tested	−5.1	−4.5

Eight weeks after the commencement of the corticosteroid (the total amount of corticosteroid received was equivalent to 5,193 mg of prednisone), she developed severe and progressive shortness of breath, which rapidly evolved into type I respiratory failure (Figure 2). Blood results are shown in Table 1. A CT scan of the thorax revealed right pleural effusion and bilateral infiltrates and cavitation (Figure 1B). A blood culture, pleurocentesis, and bronchoscopic examination were performed. The pleural fluid and bronchoalveolar lavage samples were sent for bacterial and fungal culture. The bronchoalveolar lavage sample was also sent for NGS analysis. Empirical intravenous meropenem, vancomycin, and micafungin were started.

After 3 days of incubation, the aerobic bottles of two sets of blood cultures were positive for a Gram-positive rod which was also modified Ziehl-Neelsen stain positive (Figure 2). The same bacterium was also recovered from the pleural fluid as well as four bronchoalveolar lavage samples collected over a period of 6 days. MALDI-TOF MS by the direct transfer method using the MALDI-TOF MS spectrometer (Bruker Daltonik) and the spectrum analyzed with IVD MALDI Biotyper 2.3 and reference library DB-9607 (Bruker Daltonik) did not show any confident identity. 16S rDNA gene targeted NGS (tNGS) of the isolate using the Oxford Nanopore Technologies' MinION device with protocols described (8) revealed that the 16S rDNA gene sequence of the isolate (HKU-SZH_220830) was identical to those of *N. kroppenstedtii* (GenBank accession numbers NR133794.1, DQ157924.2, and MN567716.1), indicating that the isolate was a strain of *N. kroppenstedtii*. In addition, tNGS analysis of a bronchoalveolar lavage sample was also positive for *Pneumocystis jirovecii*, cytomegalovirus, *Klebsiella pneumoniae*, Epstein-Barr virus, and *Candida albicans* sequence reads. Meropenem and vancomycin were stopped and intravenous imipenem-cilastatin, amikacin, and cotrimoxazole were started. Unfortunately, despite intensive care and extracorporeal membrane oxygenation, the patient continued to deteriorate and succumbed 18 days after the development of shortness of breath.

## Discussion

In this study, we describe the first report of a patient with *N. kroppenstedtii* bacteremic pneumonia and empyema thoracis diagnosed by tNGS. *N. kroppenstedtii* was first discovered in 2014 in a bronchoalveolar lavage sample of a lung transplant recipient with pulmonary infection (case 1, Table 2) (4). Since then, three additional cases of *N. kroppenstedtii* infections have been described in the literature (cases 2, 3, and 4 in Table 2) (9–11). As for the patient in the present report (case 5, Table 2), she received a high dose corticosteroid for 2 months because of suspected adult Still's disease and developed *N. kroppenstedtii* bacteremia, pneumonia, and empyema thoracis. *N. kroppenstedtii* was recovered from two sets of blood cultures as well as her pleural fluid and four bronchoalveolar lavage samples. Including the present case, all five patients with *N. kroppenstedtii* infections had major underlying diseases [malignancies (cases 2 and 3), autoimmune diseases (cases 4 and 5), and transplant recipient (case 1)] that required immunosuppressive treatment, including a high dose corticosteroid (cases 2, 4, and 5), anti-cancer agents (cases 2 and 3), and targeted therapy (cases 2 and 5). The clinical syndromes of the five patients include pneumonia (cases 1 and 5), brain abscess (cases 2 and 3), infective endocarditis (case 2), spinal vertebral abscess (case 4), and endophthalmitis (case 3). Bacteremia was present in three patients (cases 2, 3, and 5). Despite treatment, three patients succumbed, including the present patient. Since mortality is so high, a high index of suspicion, particularly in immunocompromised patients, is crucial. Since *Nocardia* species do not replicate as rapidly as other pyogenic bacteria, the laboratory may have to be warned if such a diagnosis is suspected. For example, the blood cultures from the present patient were only positive after 62 h of incubation. Furthermore, molecular methods for direct detection of the bacterium from clinical specimens and identification should be considered for rapid diagnosis and prompt commencement of the appropriate antibiotics.

**Table 2:** Patients with *Nocardia kroppenstedtii* infection in the literature.

**Case No**.	**References**	**Sex/age**	**Underlying disease**	**Immunosuppressive treatment for underlying disease**	**Systemic involvement of *N. kroppenstedtii* infection**	**Specimen(s) from which *N. kroppenstedtii* was isolated**	**Identification method**	**Antimicrobial treatment**	**Patient outcome**
1	Jones et al. (4)	Not mentioned	Lung transplant	Not mentioned	Not mentioned	BAL	16S rDNA gene sequencing	Not mentioned	Not mentioned
2	Kilianski et al. (8)	M/72	Mantle cell lymphoma	Bendamustine, rituximab, bortezomib, ibrutinib, methotrexate, and methylprednisolone	CNS, cardiovascular system	Blood	16S rDNA gene sequencing	Meropenem, cotrimoxazole, levofloxacin, linezolid, ceftriaxone, minocycline, and ciprofloxacin	Succumbed
3	Majeed et al. (9)	F/59	Lung carcinoma	Radiotherapy, carboplatin, and etoposide	Eye, brain	Blood	Gene sequencing	Vancomycin, piperacillin-tazobactam, meropenem, and intravitreal injection of amikacin	Succumbed
4	Venkat et al. (10)	F/78	Autoimmune hemolytic anemia	Corticosteroid	Spine and spinal cord	Pus	16S rDNA gene sequencing	Cloxacillin, cotrimoxazole, and carbapenem	Improved
5	Present case	F/71	Adult Still's disease	Dexamethasone, methylprednisolone, cyclosporin A, and tocilizumab	Lung	Blood, BAL, and pleural effusion	16S rDNA target NGS	Meropenem, vancomycin, micafungin, imipenem-cilastatin, amikacin, cotrimoxazole, ceftriaxone, and tigecycline	Succumbed

BAL, bronchoalveolar lavage; CNS, central nervous system; NGS, next-generation sequencing.

16S rDNA gene amplification coupled with NGS using the Oxford Nanopore Technologies' MinION device is an alternative to 16S rDNA gene sequencing. In the clinical setting for infectious diseases, NGS is most often used for patients who have a fever without localizing features or culture-negative infections. We have recently reported its application in confirming the first case of listeria meningitis in a patient with autoantibody against interferon gamma, understanding the spectrum of Q fever, fungal infections, culture-negative meningitis and encephalitis, and Whipple disease (12–16). As for rapid diagnosis, we have confirmed the first case of *Mycobacterium marinum* infection diagnosed by NGS and the patient received prompt anti-mycobacterial therapy and recovered (17). In the present study, we demonstrated that 16S rDNA gene amplification coupled with NGS using the Oxford Nanopore Technologies' MinION device is a feasible alternative to the more traditional 16S rDNA gene sequencing which requires a Sanger sequencer. Since the equipment cost of a Sanger sequencer is high, it is not widely available in most clinical microbiology laboratories. However, the low equipment cost, short turn-around-time, and portable size of the recently invented Oxford Nanopore Technologies' MinION device have made the use of NGS within clinical microbiology laboratories feasible (18, 19). For the present patient, it has only taken 9 h to identify the culture isolate as *N. kroppenstedtii* using the Oxford Nanopore Technologies' MinION device (Figure 2). The fact that it is highly scalable also makes this technology highly adaptable to the different capacities of laboratories. Such technical advancement will have a major impact on direct NGS analysis of clinical samples as well as rapid identification of culture isolates, hence improving patient management. While NGS is a useful tool for laboratory diagnosis of infectious diseases, it is important to note that a bronchoalveolar lavage sample collected from the present patient was also positive for *P. jirovecii*, cytomegalovirus, *K. pneumoniae*, Epstein-Barr virus, and *C. albicans* sequence reads. While *P. jirovecii* could be a co-pathogen and was hence treated with a high dose of cotrimoxazole, the other microbes were considered colonizers only. As sequence reads from colonizers and/or contaminants are frequently encountered in NGS analysis, these results must be interpreted discreetly in the clinical context of the patients.

## Data availability statement

The 16S rDNA gene sequence of HKU-SZH_220830 has been deposited in GenBank (accession number: OQ975976).

## Ethics statement

Written informed consent was obtained from the patient for the publication of this case report.

## Author contributions

FX and PW wrote the manuscript. FX reviewed the clinical data. SLo supervised the microbiological investigations. YX and QL processed and analyzed the 16S rDNA gene sequence data. SLa and PW revised the manuscript. All authors have read and approved the final version of the manuscript.
